# The Influence of Preoperative Mood and Treatment Expectations on Early Postsurgical Acute Pain After a Total Knee Replacement

**DOI:** 10.3389/fpsyt.2022.840270

**Published:** 2022-04-26

**Authors:** Julia Stuhlreyer, Regine Klinger

**Affiliations:** Department of Anaesthesiology, University Medical Centre Hamburg-Eppendorf, Hamburg, Germany

**Keywords:** placebo, nocebo, treatment expectation, TKR (total knee replacement), postoperative pain, surgery, preoperative mood, mediation analysis

## Abstract

**Background:**

Reducing postoperative pain immediately after surgery is crucial because severe postoperative pain reduces quality of life and increases the likelihood that patients develop chronic pain. Even though postoperative pain has been widely studied and there are national guidelines for pain management, the postoperative course is differently from one patient to the next. Different postoperative courses could be explained by factors related to the treatment context and the patients. Preoperative emotional states and treatment expectations are significant predictors of postoperative pain. However, the interaction between emotional states and preoperative treatment expectations and their effect on postoperative pain have not yet been studied. The aim of our study was to identify the interaction between emotional states, treatment expectation and early postsurgical acute pain.

**Methods:**

In this prospective clinical trial, we enrolled patients who had received a TKR at a German hospital between October 2015 and March 2019. Patients rated their preoperative pain on a numeric rating scale (NRS) 0–10 (0 = no pain and 10 = worst pain imaginable), their emotional states preoperatively on the Pain and State of Health Inventory (PHI), their preoperative treatment expectations on the Stanford Expectation of Treatment Scale (SETS), and their postoperative level of pain on a NRS 0–10.

**Findings:**

The questionnaires were completed by 122 patients (57% female). Emotional states predict negative treatment expectation *F*(6, 108) = 8.32, *p* < 0.001, with an excellent goodness-of-fit, R^2^ = 0.31. Furthermore, a mediator analysis revealed that the indirect effects and therefore relationship between the emotional states sad (ab = 0.06, 95% CI[0.01, 0.14]), anxious (ab = 0.13, 95% CI[0.04, 0.22]), and irritable (ab = 0.09, 95% CI[0.03, 0.17]) and postoperative pain is fully mediated by negative treatment expectations. Whereas the emotional states tired (ab = 0.09, 95% CI[0.03, 0.17]), dizzy/numb (ab = 0.07, 95% CI[0.01, 0.20]), weak (ab = 0.08, 95% CI[0.03, 0.16] are partially mediated by negative treatment expectations.

**Conclusion:**

The relationship between emotional states and postoperative pain is mediated by negative treatment expectations. Therefore, innovative treatment strategies to reduce postoperative pain should focus on eliminating negative treatment expectation through establishing a differentiated preoperative expectation management program that also focuses on emotional states.

## Introduction

Postoperative pain is often still treated inadequately (1–4). Many patients report moderate to severe pain after surgery, which results in stress and inhibits postoperative recovery (1, 5, 6). Furthermore, severe postoperative pain can result in chronic pain, decreased quality of life, and an increased need for opioids and other analgesics, which can lead to the abuse of analgesics (5). The misuse of opioids has contributed to the opioid crises in the United States, which is having devastating consequences for the parties concerned and the health care system (7). To decrease the potential negative short- and long-term effects, the optimization of postoperative pain management is necessary and highly relevant.

The total knee replacement (TKR) is a surgical procedure that is associated with severe postoperative pain (8). Specifically, 58% of the patients who undergo a TKR experience moderate to severe pain directly after the surgery (8). Postoperative pain of TKRs is generally treated with analgesics (9). However, the pain experience of patients who undergo TKR surgery and have the same postoperative medical treatment differs significantly from one individual to the next (10). The significant difference in pain ratings, despite identical treatment, emphasizes the difficulty in optimizing pain treatment. Individual differences can be explained by the fact that the combination of biological, psychological, and social aspects influences the experience of pain (11). Hence, different postoperative courses could be explained by influencing factors related to the patients or the treatment context. Especially the mechanisms “catastrophizing,” “anxiety,” “depression,” and “focus on pain” influence pain processing negatively (12). In addition, the processing of pain is significantly influenced by the psychological aspect “expectation” (13), which has been widely studied in placebo and nocebo research. Positive expectations of the surgery and treatment outcomes can have a significant positive impact, while negative treatment expectations can suppress endogenic analgesic processes. Expectations are important because they interact with the endogenous opioid system, which, subsequently, relieves pain (14, 15). Therefore, expectations as a mechanism for pain relief should be considered for postoperative pain treatment.

In the long term, 20% of patients who received a TKR experience pain for 1 year after the surgery (16, 17). However, the exact process underlying the transition from acute pain to chronic pain is still unknown (18). In this regard, it is known that early severe postoperative pain and psychological aspects and expectations in particular play important roles in pain processing (19). To reduce the probability of a transition from acute postoperative pain to chronic pain, it is essential to influence expectations positively preoperatively and to control postoperative pain directly after the surgery. Therefore, it is highly necessary to detect the underlying mechanisms of early acute postsurgical pain.

To reduce early postoperative pain, potential predictors must be identified and treated. Significant predictors for postoperative pain are preoperative emotional states (e.g., anxiety, depression) and preoperative treatment expectations. Hence, not only postoperative pain processing mechanisms are relevant for the control of postoperative pain, but it can also be predicted and, potentially, influenced preoperatively. Higher pre-operative anxiety ratings lead to increased pain during the time in the ward and at home (20, 21), and an increased hospital stay (22) after TKR surgery (23). Preoperative anxiety and depression in patients scheduled for a total knee arthroplasty (TKA) has been associated with a higher level of knee disability (24). Equally, preoperative anxiety and depression increase postoperative pain (25) and the need for analgesics for patients undergoing a TKR (26). In addition, more severe depression is associated with an increase in postoperative complications (27). Postoperative pain and postoperative recovery of patients receiving a TKA (28) are influenced by emotional states and treatment expectations (29). Treatment expectations can be specifically understood (e.g., “the analgesics will help to reduce the postoperative pain”) or can be rather vague (e.g., “the treatment will help me”). To understand the underlying mechanisms and to establish adequate innovative pain treatment methods, it is relevant to understand the combined effect of the relationship and interaction between preoperative emotional states and treatment expectations related to early postoperative pain.

To our knowledge, this is the first time that the relationship and interaction between preoperative emotional states and treatment expectations relating to early postoperative pain in patients receiving a TKR has been analyzed. Therefore, the aim of this paper is to investigate the relationship between preoperative emotional states and treatment expectations related to early postoperative pain. Specifically, we aimed to investigate whether preoperative emotional states have a direct influence on early postoperative pain or whether postoperative pain is influenced by treatment expectations. We expected that treatment expectations play a mediating role between emotional states and postoperative pain.

## Materials and Methods

### Study Populations

Patients were eligible if they were at least 18 years old and received a TKR due to knee osteoarthritis and excluded if they were cognitively impaired, had an insufficient command of the German language, suffered from mental disorders (according to ICD-10; except F45.41), consumed any mind-altering substances (e.g., psychoactive drugs, including illegal drugs), or suffered from pain requiring special causative medical treatment (e.g., cancer-related pain). Patients were only included if they received a primary TKR. Patients who received a replacement for an existing protheses were excluded. All participation was voluntary, the patients were informed about the study and provided informed written consent.

### Materials

In this prospective clinical trial, we enrolled patients who received a total knee replacement (TKR) due to osteoarthritis at a German hospital between October 2015 and March 2019. All patients who met the inclusion criteria and had none of the exclusion criteria were invited to participate in the study. The relevant instruments are scales to measure emotional states, treatment expectations, and postoperative pain.

#### Emotional States

Patients rated their emotional states with the Pain and State of Health Inventory (30). The instrument provides good internal consistency and is validated in the context of perioperative care for patients receiving a TKR (30). This instrument is mainly used to evaluate the course of postoperative recovery related to preoperative ratings. In this study, we specifically investigated preoperative emotional states ratings. The emotional states include the items being “sad,” “anxious,” “weak,” “irritated,” “numb/dizzy,” and “tired” and were measured on a numerical rating scale (NRS) 0–10 (0 = not at all; 10 = very anxious, weak etc.).

#### Expectations

There are different options for measuring treatment expectations in clinical studies in the perioperative setting (29). Treatment expectations are always manifold and can, *inter alia*, include general treatment expectations (e.g., “I expect good outcomes from medical treatment”) or specific expectations related to the treatment or a symptom (e.g., “I expect bearable pain after the surgery”). However, the majority of generally utilized expectation measures used in clinical studies, if expectations are included in the study design, are not validated single scales that depend on the research question. The only treatment expectation scales validated thus far in the perioperative setting is the Stanford Expectation Treatment Scale (SETS) (31). The SETS can be divided into positive and negative treatment expectations and assesses corresponding scales of positive and negative expectations of the planned treatment on a 7-point Likert scale, with additional open questions about the planned treatment and expected benefits or negative side-effects. Example items for the positive treatment expectation are “the treatment will be completely effective,” with answer options ranging from strongly disagree = 1 to strongly agree = 7, and an example of a negative treatment expectation item is “I am worried about my treatment,” with the response options also ranging from strongly disagree to strongly agree. High scores in positive treatment expectation of SETS indicate that patients do not expect that their treatment will be successful, nor that the treatment will improve symptoms. Whereas low scores in positive treatment indicate that patients expect that their treatment will be successful and improve their symptoms. In contrast, high scores in negative treatment indicate that patients are not worried about the treatment. Whereas low scores in negative treatment expectation indicate that the patients are worried about their treatment and that they are nervous about possible negative treatment effects, The patients completed the SETS 1 day prior to surgery to measure their treatment expectations of the TKR.

#### Preoperative Pain

To measure preoperative pain, patients were asked 1 day prior the surgery to rate their pain on a NRS 0–10 (0 = no pain; 10 = most pain imaginable).

#### Postoperative Pain

To measure postoperative pain, patients were asked to rate their pain on an NRS 0–10. Patients rated their pain every 2 h on the first day after the surgery, from 6 am until midnight, in a pain diary. Subsequently, the mean pain rate for the day was calculated from the pain ratings.

### Study Design

In this prospective clinical trial, we investigated the relationship between preoperative emotional states and treatment expectations and their effect on postoperative pain for patients receiving a TKR. All patients who received a TKR at the German hospital center, Schön Klinik Hamburg Eilbek, were screened on paper by a study physician. If the patients met the inclusion criteria on paper, they were screened in person by the study physician 1 day prior to the surgery. If the patients met the inclusion and none of the exclusion criteria, they were informed one day prior to their surgery and asked to participate. All patients participated voluntarily and provided written consent. To assess their emotional states and preoperative treatment expectations on the relevant scales, patients completed the PHI (30) and the SETS (31) 1 day prior to surgery. To measure the influence of these ratings on postoperative pain, patients rated their postoperative level of pain on an NRS 0–10 (0 = no pain and 10 = worst pain imaginable) 1 day after the surgery. Preoperative pain assessed 1 day prior the surgery on a NRS 0–10 will be included as a possible confounder. These were all self-ratings and, therefore, subjective. They were completed by the patients without the presence of a researcher.

### Statistical Analysis

All data were entered into SPSS and double-checked by two researchers independently. The analyses were performed with the statistical package IBM SPSS Statistics (version 27.0; IBM Inc., Armonk, NY, United States). The tests with *p* < 0.05 were considered statistically significant. In the path model, the independent variables were the individual emotional states, the dependent variable was postoperative pain, the mediators were treatment expectations of medical treatment, and the confounder was preoperative pain. To calculate the path model (model 4) of the SPSS Hayes’ macro, PROCESS (32), which uses ordinary least square regression and yields unstandardized path coefficients for total, direct, and indirect effects, was applied. Bootstrapping with 5,000 samples together with heteroscedasticity consistent errors were employed to compute the confidence intervals and inferential statistics. Effects were deemed significant when the confidence interval did not include zero. The interpretation of the goodness-of-fit varies between research fields. We applied the interpretation according to Cohen (33), in which an adjusted |R^2^| = 0.02 indicates a weak, |R^2^| = 0.13 a mediate, and |R^2^| = 0.26 a high goodness-of-fit for the overall model. Missing values were not completed, because the missing data was assumed to be random.

#### Sample Size

The sample size was estimated by using Table 3 of Fritz and Mackinnon (34). The table summarizes simulations for a power of 0.8 with a significance level of *p* < 0.05 to assess the required sample size for mediation effects. PROCESS is based on the bootstrapping method to analyze mediation effects. Hence, the percentile bootstrapping row in the table is relevant for our sample size calculations. Former research found a medium association between emotional states and treatment expectation (α-path) and a large relationship between treatment expectation and postoperative pain (β-path) (35, 36). The estimated size of the α-path is 0.26 and the estimated size of the β path is 0.59. Hence, 122 patients were required to assess the mediation effect for our study design.

## Results

### Demographic Characteristics of Participants

**FIGURE 1:**
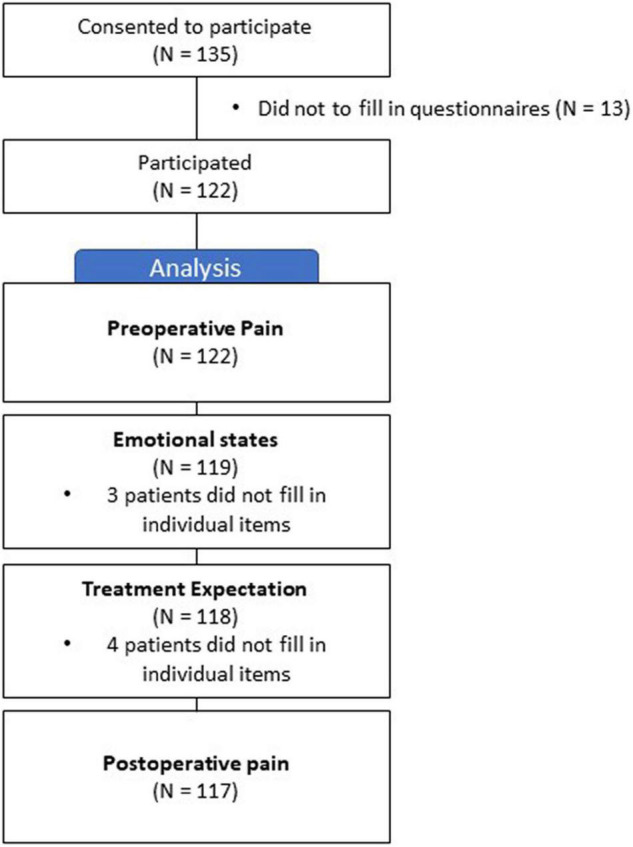
Inclusion flow chart.

A total of 122 patients participated in the study (Figure 1). Of the participants, 70 (57%) were female and 52 (43%) were male. The average age of the participants was 68 years (SD = ± 9.4), and 66% of the patients were married. For more detailed information, please refer to Table 1.

**TABLE 1:** Basic information on participants.

Characteristic	Total sample
Sample size	122
Sex – female (%)	70 (57.4)
**Age (years) (%)**	
18 – 40	0 (0)
41 – 50	6 (4.9)
51 – 60	19 (15.6)
61 – 70	42 (34.4)
71 – 80	48 (39.3)
81– 88	7 (5.7)
Mean age (years) (SD)	67.96 (9.4)
**Marital status (%)**	
Unmarried	10 (8.2)
Married	80 (65.6)
Divorced	11 (9.0)
Widowed	17 (13.9)
Stable partnership	3 (2.5)
Missing value	1 (0.8)
**Education (%)**	
No educational qualification	1 (0.8)
Lower secondary school	51 (41.8)
Intermediate secondary school	42 (34.4)
High school/A-levels	11 (9.0)
College or beyond	16 (13.1)
Missing value	1 (0.8)

*Data may not total 100% because of rounding.*

### Emotional States

A total of 119 patients completed all items related to emotional states. The internal consistency of emotional states in this sample was Cronbach’s α = 0.83. Preoperatively, the mean rating of being sad on an NRS 0–10 (0 = not sad; 10 = very sad) was 2.05 (SE = 0.20; 95% CI [1.64, 2.45]). The mean rating of being anxious was on an NRS 0–10 (0 = not anxious; 10 = very anxious) was 2.31 (SE = 0.23, 95% CI [1.85, 2.76]). The mean rating of being tired on an NRS 0–10 (0 = not tired; 10 = very tired) was 2.45 (SE = 0.20, 95% CI [2.05, 2.85]. The mean rating of being numb/dizzy on an NRS 0–10 (0 = not numb/dizzy; 10 = very numb/dizzy) was 0.94 (SE = 0.16; 95% CI [0.62, 1.25]). The mean rating of being weak on an NRS 0–10 (0 = not weak; 10 = very weak) was 1.97 (SE = 0.21; 95% CI [1.56, 2.39]). The mean rating of being irritated on an NRS 0–10 (0 = not irritated; 10 = very irritated) was 1.61 (SE = 0.19, 95% CI [1.24, 1.98]).

### Treatment Expectations

A total of 118 patients completed all items related to positive and negative treatment expectations. The mean negative treatment expectation was 4.77 (SD = 1.43), which implies that, on average, patients neither agreed nor disagreed or slightly disagree that they expected a negative treatment outcome. The internal consistency of this sample for negative treatment expectation was Cronbach’s α = 0.78. The mean rating for positive treatment expectation was 2.29 (SD = 0.85) which implies that, on average, patients agree slightly to moderately on expecting positive effects from their treatment.

### Preoperative Pain

All patients rated their preoperative pain. The mean preoperative pain score on the NRS 0–10 1 day prior the surgery was 6.5 (SE = 2.00; 95% CI [6.20, 6.89].

### Postoperative Pain

A total of 117 patients rated their postoperative pain. The mean postoperative pain score on the NRS 0–10 on the first day after the surgery was 4.74 (SE = 0.16; 95% CI [4.42, 5.05]). The internal consistency for repeated measured level of pain of this sample was Cronbach’s α = 0.84.

### Regression

#### Negative Treatment Expectations

The results show that emotional states predict negative treatment expectation *F*(6, 108) = 8.32, *p* < 0.001, with excellent goodness-of-fit R^2^ = 0.31. The results reveal that preoperative pain does not confound the regression. Moreover, negative treatment expectation predicts worse postoperative pain *F*(1,111) = 7.65, p = 0.007, with a weak goodness-of-fit R^2^ = 0.06. Being anxious (ß = −0.54), dizzy/numb (ß = −0.13), and irritated (ß = −0.13) are the strongest factors influencing negative treatment expectations, while being tired (ß = 0.05) and feeling weak (ß = −0.02) are the least influential predictors. A feeling of anxiety (*t* = −2.56, *p* = 0.01), being dizzy/numb (*t* = −2.64, *p* = 0.01), and irritated (*t* = −2.53, *p* = 0.01) had a statistical significantly influence on negative treatment expectations.

#### Positive Treatment Expectations

The results show that emotional states do not predict positive treatment expectation *F*(6,108) = 1.94, *p* = 0.08) with a mediate goodness-of-fit adjusted R^2^ = 0.10. Furthermore, positive treatment expectation does not predict postoperative pain *F*(1,112) = 1.09, *p* = 0.30) with no goodness-of-fit adjusted R^2^ = 0.001.

### Mediator Analysis

#### Negative Treatment Expectations

**FIGURE 2:**
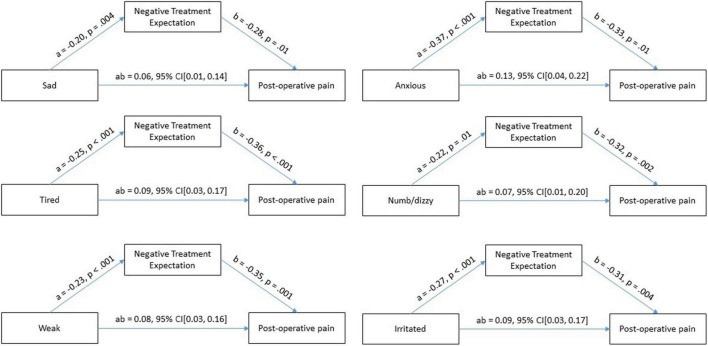
Test of mediation effect of treatment expectation on emotional states and postoperative pain.

After the mediator (negative treatment expectation) and the confounder (preoperative pain) were entered into the model, the emotional states sad (*a* = −0.20, *p* = 0.004), anxiety (*a* = −0.37, *p* < 0.001), tired (*a* = −0.25, *p* < 0.001), dizzy/numb (*a* = −0.22, *p* = 0.01), weak (*a* = −0.23, *p* < 0.001), and irritated (*a* = −0.27, *p* < 0.001) predicted the mediator significantly, which, in turn, predicted postoperative pain significantly (see Figure 2). We found that the relationship between preoperative emotional states (feeling sad, anxious, tired, dizzy/numb, weak, and irritated) and postoperative pain was fully mediated by negative treatment expectations. The direct effect (c’) of the emotional states (sad, anxious, and irritated) to postoperative pain was not significant. Whereas the direct effect of the emotional states (tired, numb/dizzy, and weak) was significant. The direct effects were sad (*c’* = −0.04, *p* = 0.64), anxious (*c’* = −0,07, *p* = 0.36), tired (*c’* = −0,20, *p* = 0.01), numb/dizzy (*c’* = −0.20, *p* = 0.04), weak (*c’* = −0.19, *p* = 0.02), and irritated (*c’* = −0.1, *p* = 0.17), while the indirect effects for emotional states were sad (*ab* = 0.06, 95% CI[0.01, 0.14]), anxious (*ab* = 0.13, 95% CI[0.04, 0.22]), tired (*ab* = 0.09, 95% CI[0.03, 0.17]), feeling numb/dizzy (*ab* = 0.07, 95% CI[0.01, 0.20]), weak (*ab* = 0.08, 95% CI[0.03, 0.16]), and irritated (*ab* = 0.09, 95% CI[0.03, 0.17]). This implies that the interaction between emotional states (sad, anxious, irritated) and postoperative pain are fully mediated and the emotional states (tired, numb/dizzy, and weak) are partially mediated by negative treatment expectation.

## Discussion

In the present study, we provide evidence that negative expectations are an important mediator for postoperative pain. Negative treatment expectations correlates with the emotional states of feeling sad, anxious, tired, numb/dizzy, weak, and irritated. This aligns with previous research that also found evidence that preoperative mental states influence postoperative pain (36). Furthermore, our findings demonstrate that individual emotional states correlates with negative treatment expectations. However, the examination of the prognostic effect of the combined investigated emotional states on negative treatment expectations and their prediction of negative treatment expectation showed that especially anxiety, numbness/dizziness, and irritability significantly predict negative treatment expectations. Interestingly, postoperative pain is mediated through negative treatment expectations, which can be interpreted that, to reduce postoperative pain, a treatment that focuses on negating negative treatment expectations is necessary and highly relevant.

Our results align with the bio-psycho-social pain model that holds that pain experience is shaped by somatic, psychological, and social factors (11). In our study, we focused on perioperative psychological factors, which is a bio-psycho-social context. We also found that psychological factors, such as emotional states that influence treatment expectations, further influence the experience of pain. Previous research has also shown that psychological factors influence pain experience and pain-related impairment in patients with joint degeneration (37–39). The focus of studies that investigate psychological factors is usually on depressive symptoms (40, 41). Other research has shown that postoperative pain experienced by patients who undergo a TKR is influenced by the severity of the preoperative pain, pain catastrophizing, depression, and pain-related impairment (42). Furthermore, one study discovered that preoperative catastrophizing and a lack of coping strategies predict a higher postoperative pain level (43). Our study adds to the knowledge by showing that patients do not necessarily have to show explicit depressive symptoms, but any emotionally impaired state can influence treatment expectations and, subsequently, postoperative pain.

Our findings suggest that emotional states predict treatment expectations and, therefore, play a critical role in the treatment outcome for postoperative pain. According to the literature concerning placebo mechanisms, positive treatment expectations are crucial to enhance surgical treatment outcomes (44, 45). However, underlying mechanisms have to be uncovered to be able to influence treatment expectations. The specific underlying mechanisms relating to our results can only be speculated so far. One possibility could be that the nocebo system is activated, and the placebo system deactivated. This would imply that, on the one hand, due to the activated nocebo system, biochemical changes occur through the activation of cholecystokinin that facilitate pain transmission (46), and the dopaminergic system and opioid release may also be deactivated as a consequence (47). On the other hand, it is also possible that the placebo system is deactivated. The placebo system is significantly influenced by the activation of the dopaminergic system and the release of endorphins and endogenic opioid system (48–50). In addition, selective attention could be an important modulator (51, 52), and the specific modulator and mechanisms should be investigated in further research.

Interestingly, we found that emotional states influence negative treatment expectations but not positive treatment expectations. One reason could be that the placebo and nocebo systems do not share the same network (53). Nocebo effects might be produced through the medial pain system (54), with the hippocampus, the dopaminergic system, and the release of endorphins and endogenic opioids as key players. We could therefore also confirm that negative treatment expectations are not the opposite of positive treatment expectations and that patients who expect negative treatment outcomes do not automatically deny positive treatment outcomes. However, if negative expectations predominate, they could either increase the activity of the nocebo system or hinder the activity of the placebo system, so that both pathways could cause an increase in the postoperative pain experienced. Therapeutic interventions for perioperative pain management are therefore, on the one hand, the reduction of negative expectations and, on the other hand, the strengthening of the placebo system. A reduction in negative treatment expectations may be achieved through preoperative differentiated and specialized expectation management. This could be achieved in a program that focuses on individual treatment expectations, including previous negative treatment experiences and personal anxieties. Our results imply that expectation management should also include a focus on emotional states. The placebo system could be strengthened by directing all the patient’s senses toward the positives of postoperative analgesia. Patients should be aware of analgesic action, know how the medication works in their bodies, when it will take effect, and how long the effect lasts. Knowledge about the medication could provide them with insight into their pain management and, in the sense of open medication, strengthen the placebo component inherent in every analgesic (55). It is important to investigate the influence of other emotional states on positive treatment expectation in further research, which could provide more insight into how to decrease postoperative pain and how treatment expectations can be positively influenced.

### Strengths

This is the first study to report on the interaction between emotional states, expectations and postoperative pain in a clinical sample of patients expected to experience early acute postoperative pain. Previous studies have investigated healthy participants rather than a clinical sample. However, pain pathways, experiences, and treatment expectations may differ between healthy and clinical participants. With this study, we detected that emotional states, such as being sad, anxious, feeling dizzy/numb, weak or irritated, individually or in combination, can influence negative treatment expectations, which, in turn, influenced postoperative pain experiences in a clinical sample. We further detected that the relationship between emotional states and postoperative pain is fully mediated by negative treatment expectations. Therefore, we detected the mediator between emotional states to postoperative pain in the underlying mechanisms of the placebo and nocebo effects. We further found that negative interaction between emotional states and postoperative pain is fully mediated by negative treatment expectations. In consequence, our results provide the foundation for future innovative treatment options that are required for optimal postoperative pain management.

### Limitations

Several limitations in this study warrant comment. First, the data used were based on self-reporting, and there might be altered response behavior. However, because pain is subjective and there are no objective measurement tools to assess it, postoperative pain can only be assessed *via* a self-report. Second, only patients who received a TKR were included in the study, and TKRs are often associated with severe postoperative pain. In addition, patients have usually experienced pain for a long period prior to the TKR and have often been treated conservatively. Hence, the results cannot be generalized to other surgeries *per se*, but they are a good indicator for patients undergoing TKR surgery. Patients were included, when they received a TKR due to osteoarthritis. Osteoarthritis can be classified based on the Kellgren and Lawrence system of classification (56). In this study, we did not assess the grade of osteoarthritis and can therefore not analyze if the grade of osteoarthritis influences emotional states, treatment expectations, or postoperative acute pain. Furthermore, treatment expectations were only vaguely assessed, and, therefore, no conclusions can be drawn about the relationship between symptom-specific expectations relating to postoperative pain and emotional states. However, a validated tool was used to assess treatment expectations. Due to the aim to gain a first overview about the relationship between emotional states, treatment expectations and postoperative acute pain, not many confounders were included into the study. However, treatment expectation and placebo effects and their influence on postoperative pain is complex. Hence, in further studies possible confounders (e.g., catastrophizing, depression) should be included into the study design.

### Outlook

As already noted, this study provides a foundation for future research. Resulting from our findings, there are several aspects that should be investigated. First, modulators should be investigated to discover the underlying mechanisms and the interaction between emotional states and treatment expectations. In this context, the underlying biochemical and neural mechanisms should be examined to establish their effect on perioperative procedures. Second, further studies should investigate how emotional states can be positively influenced and whether this will decrease negative treatment expectations, which should, in turn, decrease postoperative pain. Therefore, it should be investigated how negative expectations can be mitigated or changed into positive expectations. In this regard, it is necessary to further investigate the different modulators of positive and negative treatment expectations. This could be a precursor for innovative pre- and postoperative treatment strategies that can be developed to enhance treatment outcomes and the associated placebo effect. Therefore, future interventions could focus on reducing negative treatment expectations by considering the influential mechanisms of impaired emotional states. By focusing on the influential factors of negative treatment expectations, postoperative pain can be reduced directly after the surgery. Hence, developing a preoperative differentiated and specialized expectation management program will be highly relevant. In the light of the current opioid crisis (7, 57), it would be especially relevant to investigate the relationship between emotional states, treatment expectations, and analgesic consumption.

## Conclusion

This study investigated a sample of patients who received a TKR at a German hospital. The results reveal that the relationship between impaired emotional states and postoperative pain is fully mediated by negative treatment expectations. Therefore, novel and innovative treatment strategies to reduce postoperative pain should focus on negative treatment expectations through a differentiated and specialized preoperative expectation management program that should also aim to reduce the emotional states of being sad, anxious, numb/dizzy, tired, weak, and irritated. A specialized treatment expectation management program developed with consideration of our findings might mitigate and change negative expectations to influence postoperative pain positively.

## Data Availability Statement

The raw data supporting the conclusions of this article will be made available by the authors, without undue reservation.

## Author Contributions

JS and RK contributed to conception, design of the study, and wrote the first draft of the manuscript. JS performed the statistical analysis. Both authors contributed to manuscript revision, read, and approved the submitted version.

## Conflict of Interest

The authors declare that the research was conducted in the absence of any commercial or financial relationships that could be construed as a potential conflict of interest.

## Publisher’s Note

All claims expressed in this article are solely those of the authors and do not necessarily represent those of their affiliated organizations, or those of the publisher, the editors and the reviewers. Any product that may be evaluated in this article, or claim that may be made by its manufacturer, is not guaranteed or endorsed by the publisher.
